# Incidence of cerebral metastases in patients treated with trastuzumab for metastatic breast cancer

**DOI:** 10.1038/sj.bjc.6601970

**Published:** 2004-07-20

**Authors:** A J Clayton, S Danson, S Jolly, W D J Ryder, P A Burt, A L Stewart, P M Wilkinson, R S Welch, B Magee, G Wilson, A Howell, A M Wardley

**Affiliations:** 1 Departments of Medical and Clinical Oncology, Christie Hospital NHS Trust, Wilmslow Road, Withington, Manchester, M20 4BX, UK

**Keywords:** trastuzumab, brain metastases, breast cancer

## Abstract

Trastuzumab is an effective treatment for patients with metastatic breast cancer (MBC) that overexpresses HER-2. A high incidence of brain metastases (BM) has been noted in patients receiving trastuzumab. A retrospective chart review was conducted of 100 patients commencing trastuzumab for metastatic breast cancer from July 1999 to December 2002, at the Christie Hospital. Seven patients were excluded; five patients developed central nervous system metastases prior to starting trastuzumab, and inadequate data were available for two. Out of the remaining 93 patients, 23 (25%) have developed BM to date. In all, 46 patients have died, and of these 18 (39%) have been diagnosed with BM prior to death. Of the 23 patients developing BM, 18 (78%) were hormone receptor negative and 18 (78%) had visceral disease. Univariate analysis showed a significant association between the development of cerebral disease and both hormone receptor status and the presence of visceral disease. In conclusion, a high proportion of patients with MBC treated with trastuzumab develop symptomatic cerebral metastases. HER-2-positive breast cancer may have a predilection for the brain, or trastuzumab therapy may change the disease pattern by prolonging survival. New strategies to address this problem require investigation in this group of patients.

The human epidermal growth factor receptor family (HER 1–4) is involved in the control of cell growth and differentiation. Amplification of the HER-2 gene leads to overexpression of the receptor, which has been implicated in oncogenic transformation and tumour genesis. The HER-2 receptor dimerises with other members of the HER family to initiate cell signalling cascades, leading to increased cell growth and division. Transfection of the HER-2 gene into human tumour cell lines leads to the development of a more aggressive tumour cell phenotype (Neve *et al*, 2001; Rubin and Yarden, 2001). Amplification of the HER-2 gene with overexpression of the receptor is detected in 20–30% of breast cancers (Slamon *et al*, 1987, 1989) and is associated with a worse prognosis compared to those with HER-2-negative tumours (Slamon *et al*, 1987; Emi *et al*, 2002). Whether HER-2 is predictive for response to chemotherapy, and to hormonal therapy, remains controversial (Fournier *et al*, 2002). There is no controversy about the fact that HER-2 overexpression predicts the response to trastuzumab. Trastuzumab (Herceptin™) is a recombinant humanised monoclonal antibody against the HER-2 receptor. Trastuzumab administered either alone or in combination with cytotoxic chemotherapy is being used increasingly in clinical practice. Trastuzumab monotherapy is associated with response rates of 26% in the first-line setting for metastatic disease (Vogel *et al*, 2002), and 15% in patients previously treated with chemotherapy (Cobleigh *et al*, 1999). In combination with chemotherapy, trastuzumab therapy is associated with response rates of up to 70–80% (Seideman *et al*, 2001). Trastuzumab prolongs both the response duration and survival of patients with tumours overexpressing HER-2, when administered with chemotherapy compared with chemotherapy alone (Slamon *et al*, 2001).

It has recently been reported that trastuzumab levels in the CSF following intravenous infusion are 300-fold lower than serum levels, implying that i.v. trastuzumab is unlikely to be effective against CNS disease (Pestalozzi and Brignoli, 2000). Unfortunately, the prognosis of patients following the development of CNS disease is generally poor, with median survival times reported around 4–6 months. Historical data indicate that the incidence of symptomatic brain metastases (BM) in patients with metastatic breast cancer is around 10% (DiStefano *et al*, 1979; Stewart *et al*, 1981; Tsukada *et al*, 1983). However, a large post mortem study has reported the presence of central nervous system disease in up to 29.6% of patients dying from breast cancer (Tsukada *et al*, 1983). These two observations lead inevitably to the hypothesis that the prolongation of survival of patients with metastatic breast cancer using therapies which do not penetrate the CNS will result in a higher incidence of clinically overt cerebral metastases than has hitherto been observed.

A preliminary report from this institution indicated that a high proportion of patients receiving trastuzumab therapy were observed to develop cerebral metastases (Wardley *et al*, 2002).

The aim of this study was to ascertain, in a larger patient cohort, the proportion of patients receiving trastuzumab who develop cerebral metastases, and to explore whether any clinico-pathological correlates could be identified.

## METHODS

Using pharmacy records, all patients commencing trastuzumab at the Christie Hospital up until the end of December 2002 were identified. A retrospective review of the case notes extracted information on the dates of initial diagnosis of breast cancer; details of histology including oestrogen receptor (ER), progesterone receptor (PR) and HER-2 status by immunohistochemistry (CB11 antibody); each episode of disease progression; details and dates of all therapy for breast cancer, including recording sites of disease, and best responses to therapy in those sites. In all patients with a diagnosis of cerebral metastases, the CNS disease presentation was symptomatic, and was confirmed radiologically by CT or MRI scanning.

Univariate analysis has been performed to determine the potential influence on the time for the development of BM from the initiation of trastuzumab therapy of the following factors: ER status; presence of visceral (parenchymal lung or liver) metastases at the start of trastuzumab therapy; previous neoadjuvant or adjuvant chemotherapy; trastuzumab given with or without chemotherapy; number of prior systemic therapies for metastatic disease; and time from diagnosis to the development of MBC. The primary outcome analysed was the time from the start of trastuzumab to the development of BM. Cases that did not develop BM were censored at their time of death or last follow-up as appropriate. Freedom from BM curves was estimated using the Kaplan–Meier method and univariate comparisons between subgroups were made using the log-rank test. Plots and test results were obtained using the S-Plus computer software. This study was conducted in accordance with local ethical guidelines.

## RESULTS

### Study population

In all, 100 patients have been treated with trastuzumab for metastatic breast cancer at the Christie hospital from October 1999 to December 2002. Two patients with inadequate follow-up documentation and five patients diagnosed with CNS metastases prior to the start of trastuzumab were excluded from analysis. The characteristics of the 93 patients in the study population are shown in Table 1

**Table 1:** Characteristics of patients with metastatic breast cancer treated with trastuzumab

Number in study group	93
Median age (range)	48 (23–76)
	
*HER2 immunohistochemistry*	
2+	6
3+	87
	
*Hormone receptor status*	
ER+	41 (44%)
ER	52 (56%)
ER	47 (51%)
ER	5 (5%)
	
*Trastuzumab given*	
Alone	30 (32%)
With chemotherapy	53 (57%)
With hormonal therapy	8 (9%)
With chemotherapy and hormonal therapy	2 (2%)
	
*Line of therapy for trastuzumab*	
First systemic therapy	44 (47%)
Second systemic therapy	31 (33%)
Third line systemic therapy	18 (19%)
	
*Disease distribution at the start of trastuzumab therapy*	
Visceral	51 (55%)
Nonvisceral	42 (45%)
	
*Response to trastuzumab*	
Stable disease or better	53 (57%)
PD	33 (35%)
Not assessed/not assessable	7 (8%)

.

HER-2 expression by immunohistochemistry was 3+ in 87 (94%) of the patients. In all, 41 of the patients had tumours expressing ER, the remaining 52 were ER negative.

A total of 57% of the patients had a response of stable disease (SD) or better on trastuzumab. Stable disease was defined as the absence of either disease response or progression radiologically (or clinically for chest wall disease) for at least 6 months.

The median duration of trastuzumab therapy for the group was 224 days or 32 weeks (1–207 weeks). The median survival of this group of patients was 2 years.

In all, 46 patients in the group had died at the time of analysis, and the median follow-up of the patients in the group who remained alive was 10.8 months from the start of trastuzumab.

### Patients developing BM

Out of 93 patients, 23 (25%) developed symptomatic BM after commencing trastuzumab therapy. All of the 23 patients developed intracerebral metastases, with four having evidence of meningeal disease in addition. Of the 46 patients who have now died from their breast cancer, 18 (39%) were diagnosed with BM prior to death. Of the 47 patients who remained alive, five (11%) had developed CNS disease at the time of analysis.

The proportion of patients developing BM at 1 year from the start of trastuzumab was estimated to be 26.1% (95% confidence interval of 13.5–36.9%).

The median duration of trastuzumab treatment among those patients developing BM was 293 days (42–1448). The median time from the initiation of trastuzumab therapy to the development of BM was 305 days (39–933).

Following the diagnosis of BM, 16 out of 23 patients have been treated with radiotherapy to date. Out of the 23 patients developing BM, 16 went on to receive further systemic therapy. In all, 10 patients continued trastuzumab, six of these also received further cytotoxic chemotherapy (three in combination with trastuzumab, and three following trastuzumab), and four received further trastuzumab alone. Six other patients did not continue trastuzumab but received further cytotoxic chemotherapy.

**Figure 1:**
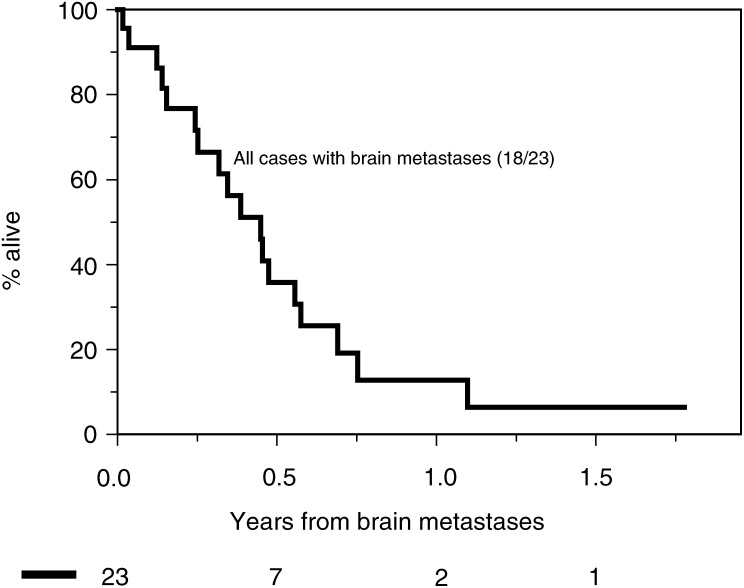
Survival of patients following diagnosis of BM.

The median survival of the patients from the diagnosis of cerebral metastases was 5.4 months (Figure 1

). The median survival of those 16 patients who went on to receive further systemic therapy was 5.6 months.

Out of the 23 patients developing CNS disease, 18 (78%) had SD or were better in sites other than the CNS in response to trastuzumab therapy. In all, 16 patients (70%) developed cerebral metastases while receiving trastuzumab therapy; in 13 out of 16 (82%), the CNS was the first site of symptomatic disease progression, and in 11 out of 16 (69%) the only site of disease progression at that time.

**Figure 2:**
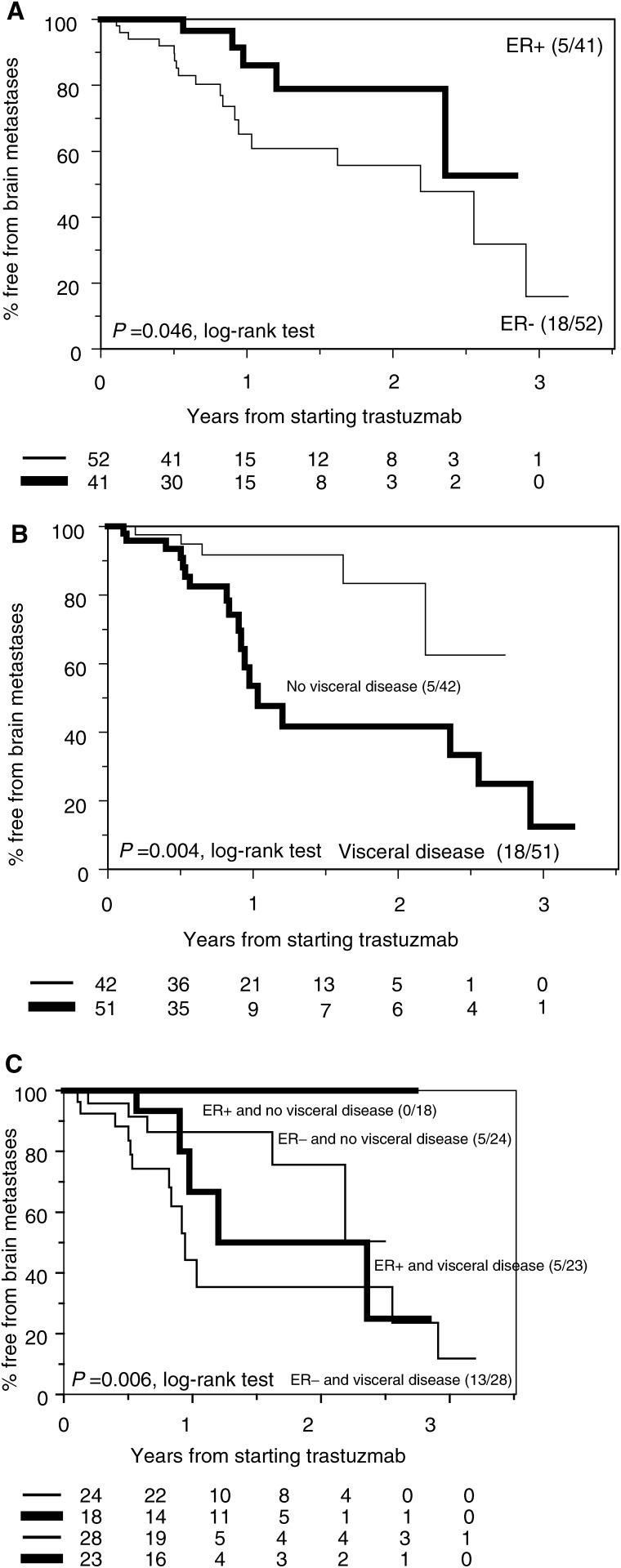
(**A**) Freedom from BM by ER status. (**B**) Freedom from BM by the presence or not of visceral metastases at the start of trastuzumab therapy. (**C**) Freedom from BM by ER status and the presence of visceral metastases at the start of trastuzumab therapy.

Five of 41 (12%) patients with ER-positive breast cancer developed BM compared to 18 out of 52 (35%) with ER-negative disease. Out of 51 patients with visceral disease at the start of trastuzumab therapy, 18 (35%) developed BM, compared with only five of 42 (12%) patients without visceral disease at the start of trastuzumab therapy. Univariate analysis showed a significant association between the time to the development of BM from the initiation of trastuzumab therapy and both hormone receptor status, and the presence of visceral disease at the start of trastuzumab therapy. This is demonstrated in Figures 2A–C

.

There was no association between development of BM and other factors analysed (i.e. neoadjuvant or adjuvant chemotherapy; whether trastuzumab was given alone or with chemotherapy; the number of prior systemic therapies for metastatic disease; or the time from initial presentation to the development of metastatic disease).

## DISCUSSION

A high proportion, 23 out of 93 (25%), of this cohort of patients with metastatic breast cancer treated with trastuzumab at a single institution, have developed symptomatic BM. Out of the 46 patients who have died, 18 (39%) have developed symptomatic cerebral metastases pre-mortem. The 95% confidence interval for the incidence of BM after 1 year of trastuzumab in this study is 13.5–36.9%, the lower limit of which is higher than the expected incidence of CNS metastases pre-mortem in historical series. The large study from Tsukada reports 1044 post mortems of breast cancer patients, with CNS disease present in 309 (29.6%), of which only 96 cases were diagnosed clinically prior to death (9%) (Tsukada *et al*, 1983).

There are other reports in the literature of a high incidence of CNS disease in patients with metastatic breast cancer treated with trastuzumab. The Dana Faber Partners Cancer Care reported CNS disease in 34% of 122 patients with metastatic breast cancer overexpressing HER-2 treated with trastuzumab (Bendell *et al*, 2003). Heinrich *et al* (2003) reported cerebral metastases in 43% of 51 patients with HER-2-overexpressing breast cancer receiving trastuzumab. Brufsky *et al* (2003), in a phase II study of docetaxel, carboplatin, and trastuzumab in 38 patients, observed that out of those patients progressing on therapy 50% developed CNS disease.

The rate of BM is particularly high in patients deriving systemic benefit from trastuzumab: 18 out of 23 (78%) patients who developed BM had SD or were better in response to trastuzumab. In the German series, 79% of the 22 patients who developed CNS disease did so while responding systemically to trastuzumab (Heinrich *et al*, 2003). In the American series, 15 of the 21 patients (71%) treated with trastuzumab as first-line therapy developed CNS metastases while still responding or having achieved SD on trastuzumab (Bendell *et al*, 2003).

The development of BM in our series was found to be significantly associated with ER negativity, and with the presence of visceral metastases at the start of trastuzumab therapy. In keeping with this, Bendell *et al* (2003) found that of patients with CNS metastases, fewer (33 *vs* 64%) had received prior hormonal therapy (a surrogate for steroid hormone receptor status), and there was a nonsignificant preponderance of visceral disease in patients who developed CNS disease. Others have also observed that patients with ER-negative disease are more likely to develop BM in comparison with patients having ER-positive tumours (Stewart *et al*, 1981; Clark *et al*, 1987; Maki and Grossman, 2000).

There are two possible nonexclusive explanations for the increased incidence of symptomatic CNS disease in patients treated with trastuzumab.

Firstly, trastuzumab therapy controls systemic disease and prolongs survival (Slamon *et al*, 2001), but does not cross the blood–brain barrier (BBB), making the CNS a ‘sanctuary site’, allowing the development of symptomatic BM which otherwise would have remained clinically silent prior to death. It has been shown that systemic administration of trastuzumab does not yield significant levels in the cerebrospinal fluid (Pestalozzi and Brignoli, 2000). There is, however, debate over whether the BBB is meaningful in cerebral metastases, and there is evidence that other agents which do not normally traverse the BBB may penetrate CNS tumour deposits (Rosner *et al*, 1986). There is only one report in the literature of a response to trastuzumab of CNS disease, and this was confounded by the use of other treatment modalities (Baculi *et al*, 2001).

Secondly, it is possible that tumours overexpressing HER-2 may have a predilection for CNS. In support of this theory, in a phase II study of docetaxel/epirubicin reporting a high incidence of CNS disease, it was found, retrospectively, that a majority of patients developing CNS disease had tumours overexpressing HER-2 (Crivellari *et al*, 2001). In addition, Miller *et al* (2003) found HER-2 overexpression to be predictive of the detection of occult CNS metastases in patients with metastatic breast cancer being screened for clinical trial entry.

Irrespective of the reason for the observed increased incidence of BM, the practical problem is that of the development of CNS disease in patients with HER-2-overexpressing tumours otherwise responding systemically to trastuzumab. Survival with BM is generally short, of the order of 4–6 months, and could potentially be prolonged if CNS disease could be controlled, or prevented. Given the association with ER-negative disease, it may be possible to further define a population within the group of patients being treated with trastuzumab who are at particularly high risk (i.e. those with ER-negative disease, and visceral metastases), although this requires verification in other studies.

There could be two approaches to this problem, both of which could potentially form the basis of a clinical trial, and there seems to be sufficient evidence in the literature to justify such a trial. One approach could be that of active prevention, with a trial of prophylactic cranial irradiation (PCI) *vs* no CNS-directed therapy for patients responding to trastuzumab therapy. Prolonged survival is seen in small-cell lung cancer (SCLC) with PCI after complete response to chemotherapy. PCI is also finding a role in non-small-cell lung cancer (NSCLC) as new treatments improve survival and reveal a higher incidence of BM (Pottgen and Stuschke, 2001), which is analogous to the prolonged survival with trastuzumab in patients with breast cancer.

An alternative approach may be one of active surveillance in patients responding to trastuzumab. It is, of course, unknown whether the early detection and treatment of CNS disease in this setting would be beneficial in terms of either survival or symptoms. With the advent of more aggressive approaches to the treatment of limited CNS disease with surgery, and radiosurgery, it is possible that this, in the context of controlled systemic disease, may be beneficial (Gerosa *et al*, 2002). Again, any such CNS ‘surveillance’ should be as part of a randomised clinical trial against no surveillance, which is currently the standard of care.

## CONCLUSIONS

Among a population of patients treated with trastuzumab, a high proportion of patients have been observed to develop BM. There may be two factors involved:

Firstly, longer survival of patients receiving trastuzumab (which does not cross the blood brain barrier) may allow the development of symptomatic BM, which otherwise would have remained clinically silent. Secondly, tumours overexpressing HER-2 may have a predilection for the CNS.

Trials of specific CNS therapy (e.g. PCI) or surveillance should be considered for these patients
